# The Effect of the Schroth Rehabilitation Exercise Program on Spinal and Feet Alignment in Adolescent Patients with Idiopathic Scoliosis: A Pilot Study

**DOI:** 10.3390/healthcare10020398

**Published:** 2022-02-20

**Authors:** Jaeyong Park, Wi-Young So

**Affiliations:** 1 Institute of Sports Health Science, Sunmoon University, 70, Sunmoon-ro 221 beon-gil, Tangjeong-myeon, Asan-si 31460, Korea; 2006076@sunmoon.ac.kr; 2 Sport Medicine Major, College of Humanities and Arts, Korea National University of Transportation, Chungju-si 27469, Korea

**Keywords:** calcaneal valgus angle, Cobb’s angle, idiopathic scoliosis, lumbar lordosis, Schroth

## Abstract

Background: This study investigated the therapeutic effects of 12-week Schroth rehabilitation exercises (SRE) in improving Cobb’s angle, scoliometer readings, lumbar lordosis, and the calcaneal valgus angle of patients with idiopathic scoliosis. Methods: This pilot study included 60 adolescent patients diagnosed with idiopathic scoliosis by a rehabilitation physician based on a Cobb’s angle of ≥10° using total anteroposterior plain radiography. Patients were classified into groups with a Cobb’s angle of 10–19° (G1), 20–29° (G2), and ≥30° (G3). Cobb’s angle, scoliometer readings, lumbar lordosis, and calcaneal valgus angles were analyzed before and after the 12-week SRE. Results: SRE improved Cobb’s angle (−6.85), scoliometer readings (−2.80), lumbar lordosis (4.23), and calcaneal valgus angles (left, −3.76; right, −2.83) regardless of the initial scoliosis angle, and within-group changes were significant (*p* < 0.001). In this study, participants in all three groups had undergone SRE, regardless of initial scoliosis severity, and the findings were significant. Conclusion: SRE can be used for patients with idiopathic scoliosis to improve asymmetric musculoskeletal morphology and the patient’s quality of life.

## 1. Introduction

Idiopathic scoliosis, for which the causes are unknown, comprises 85% of all reported cases of scoliosis. It mostly occurs during puberty, from the age of 10 through to the adolescent period, until bone growth stops [1]. At the end of the deformation process, the resultant spinal deformity causes severe functional disorders (e.g., cardiopulmonary conditions and kyphosis) [2,3,4]. Spinal deformity mainly leads to progressive thoracolumbar curvature. When the curve reaches 40°, balance issues arise in the trunk and the upper extremities are used to maintain an upright posture. At 80°, the ribs and pelvis touch each other, leading to severe pain. At a curve of >80°, pulmonary function begins to deteriorate [5]. Acting to standardize reports of non-operative research, recommendations by the Scoliosis Research Society (SRS) identify the Cobb’s angle as the primary outcome [6]. When the Cobb’s angle is >50° and physiologic deterioration is apparent (decreased lung capacity due to narrowing of the diameter of the rib cage, a decrease in proteoglycan and glycoprotein, and an increase of collagen concentration in the nucleus pulposus), surgery is considered [4]. In other cases, treatment involves conservative methods, such as spinal bracing, manipulation, physical therapy, and prescribed exercise [7,8]. Early diagnosis and treatment are therefore essential for idiopathic scoliosis [9], with early treatment involving spinal bracing, surgery, and directed exercise.

The aim of exercise therapy is to correct muscular imbalance. However, excessive and inappropriate exercise without a precise radiographic diagnosis and physical assessment may interrupt treatment and worsen the condition [10]. Schroth rehabilitation exercises (SRE) provide stable outcomes and high patient satisfaction [11,12]. SRE is a three-dimensional (3-D) exercise method that corrects scoliosis with improvement in body shape and respiratory capacity through the application of rotational breathing [13]. Similar to a compressed ball that regains its original shape through the injection of air, the SRE respiration technique applies 3-D exercise with optimum feedback to correct scoliosis curvature and deviation [14].

SRE differs markedly from the standard correction exercises that have previously been used to treat scoliosis [15,16]. The advantage of SRE is that patients can more accurately perceive their image in terms of postural imbalance due to scoliosis and improve their abnormal posture in response by intentionally changing their breathing [13]. Several studies on SRE have reported that severe spinal curvature, pain, muscular endurance, self-image, and surface topography characteristics were improved after exercise intervention [11,17,18]. Moreover, an increase in the calcaneal valgus angle negatively affects the pelvic alignment [19] and the stability of the standing posture [20]. Park et al. [21] reported a positive correlation between idiopathic scoliosis and calcaneal valgus angle. Additionally, eversion of the calcaneus significantly increases hip flexion, hip internal rotation, anterior pelvic tilt, thoracic lateral tilt, and axial rotation in the standing position [22]. This indicates that the association between the shape and position of the feet and postural alignment occurs through a functional connection between the foot and distal joints and that the closed kinetic chain mediates the movement of the lower extremities [23]. In a study of 16 children with foot deformities, Rasool et al. [24] reported an association of foot deformity with spinal abnormalities. Therefore, SRE appears to improve postural balance and reduce the curvature of scoliosis through strengthening the intercostal muscles and promoting normalization of the diaphragmatic and respiratory muscles [7].

As idiopathic scoliosis occurs spontaneously and progresses slowly, early detection and treatment are essential to reduce the requirement for surgery, alleviate psychological distress, and improve the correction rate. Early treatment using exercise is optimal for some patients [25]. In a previous study concerning idiopathic scoliosis, SRE performed in clinics under the guidance of exercise specialists significantly reduced the rate of change in Cobb’s angle and rotation angles. Compared to conventional exercises, SRE was more effective in increasing chest expansion, lung capacity, and muscle strength [11,26,27]. In addition, 8 weeks of SRE led to positive improvements in the spine angle, chest expansion, and static balance [28]. While some studies have verified the effects of SRE on Cobb’s angle [26], there have been few reports on changes in the calcaneal valgus angle and lower extremity alignment according to the degree of spinal deformity. Furthermore, we set additional goals to reduce pain and improve appearance through increasing stability in terms of controlling foot movement and biomechanically reducing postural asymmetry.

This study classified teenagers who had been diagnosed with scoliosis into three groups according to the Cobb’s angle. This study aimed to verify the effects of the application of SRE, characterized as a 3-D intervention, in the body posture by measuring and analyzing changes in Cobb’s angle, scoliometer readings, total lumbar lordosis, and calcaneal valgus angles. In addition, this study considered whether SRE improved these parameters regardless of the severity of scoliosis. Changes in scoliosis severity were reviewed in each group to assess the effectiveness of the rehabilitation program and to establish a foundation for the clinical application of SRE.

## 2. Materials and Methods

### 2.1. Participants

This study enrolled 60 teenagers who had been diagnosed with idiopathic scoliosis by a rehabilitation medicine physician. Diagnosis was based on a Cobb’s angle of ≥10° using whole spine anteroposterior plain radiographic examinations taken at Schroth Specialty Rehabilitation Clinic in Korea. Participants were classified into three groups according to Cobb’s angles of 10–19°(*n* = 20), 20–29°(*n* = 20), and ≥30°(*n* = 20) by non-random sampling, without a control group. In addition, Cobb’s angles were measured after the participants performed the SRE program for 12 weeks without dropping out during the experiment. This study included those who had been diagnosed with idiopathic scoliosis within 1 year (average of 10.2 months), and those who were considered capable of performing the exercises according to the neurological findings of their physician. Moreover, the study participants were those who were able to communicate and to run, as well as those who lived independently. All participants were encouraged to wear a brace (TSLO) during activities of daily living (ADL) after exercise. It was confirmed through consultation whether or not TSLO was to be worn during ADL before exercise. Prior to enrollment, participants had understood the purpose of the study and indicated their willingness to participate after having been provided with the details on the experimental procedures. The study participants had undergone no prior conservative treatment for scoliosis or for other orthopedic disorders and had no history of cardiovascular or pulmonary disease. This study was approved by the Institutional Review Board for Clinical Testing at Gwangju Oriental Hospital of Dongshin University (IRB no: DSGOH-017). Participants’ physical characteristics are shown in Table 1.

### 2.2. Experimental Design

**Figure 1:**
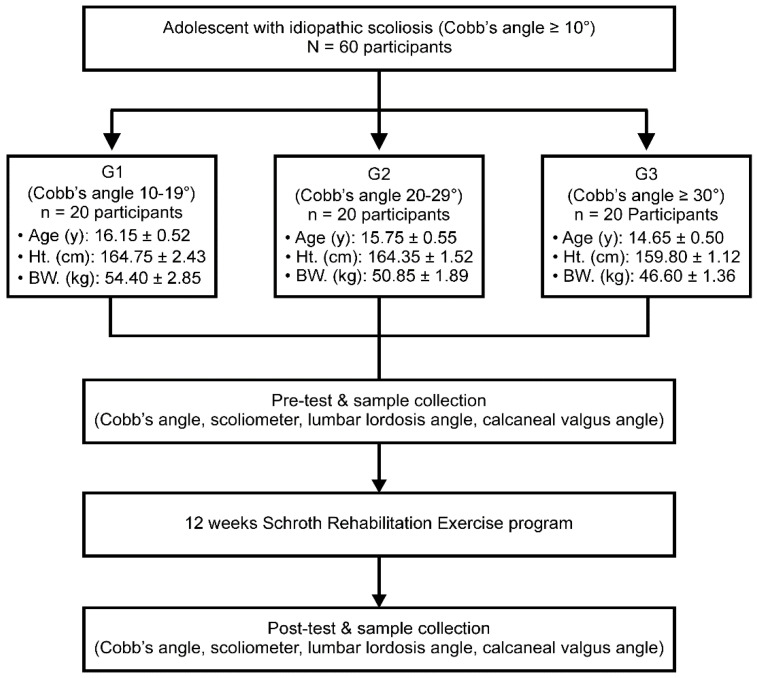
Inspection processes and procedures. G1, mild idiopathic scoliosis group with a Cobb’s angle of 10–19° (*n* = 20); G2, moderate idiopathic scoliosis group with a Cobb’s angle of 20–29° (*n* = 20); G3, severe idiopathic scoliosis group with a Cobb’s angle of ≥30° (*n* = 20); Ht., height; BW., bodyweight.

The experimental procedure of this study is shown in Figure 1.

#### 2.2.1. Measurement of the Cobb’s Angle

**Figure 2:**
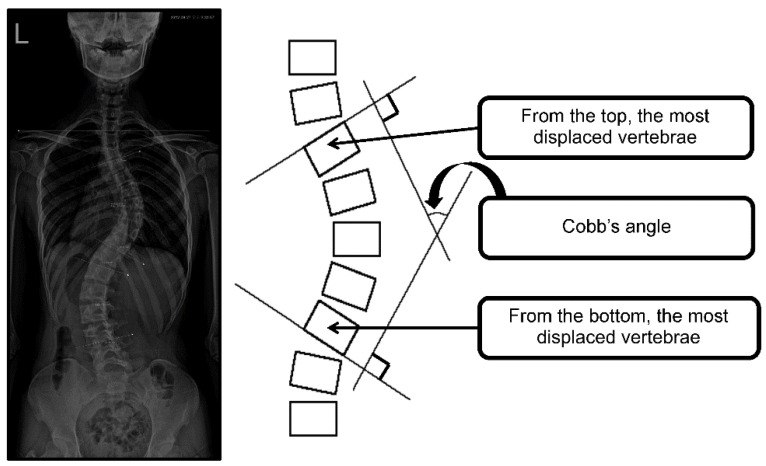
Cobb’s angle measurement.

The Cobb’s angle was measured radiographically by a rehabilitation medicine doctor. The angle between intersecting lines drawn perpendicular to the top of the top vertebrae and the bottom of the bottom vertebrae is the Cobb’s angle, as shown in Figure 2. The calculated angle determines the curvature severity [29].

#### 2.2.2. Adam’s Forward Bending Test (Scoliometer Measurement)

**Figure 3:**
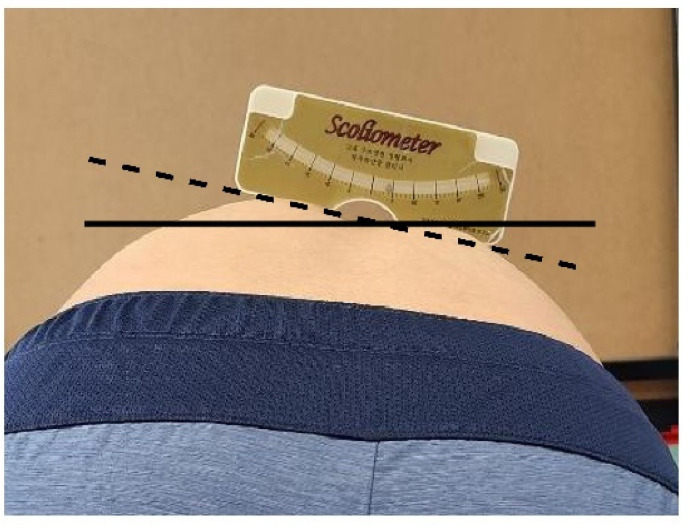
Scoliometer measurement.

To perform Adam’s forward bending test, each participant was asked to stand with their feet together, naturally relaxing both arms and hands and bending the upper body forward at 90°. The examiner stood with the participant’s back at eye level and measured the asymmetrical thoracic and lumbar rotation angles through the rib hump using a scoliometer (National Scoliosis Foundation, Watertown, MA, USA) [30]. Measurements were carried out as shown in Figure 3.

#### 2.2.3. Measurement of the Lumbar Lordosis Angle

**Figure 4:**
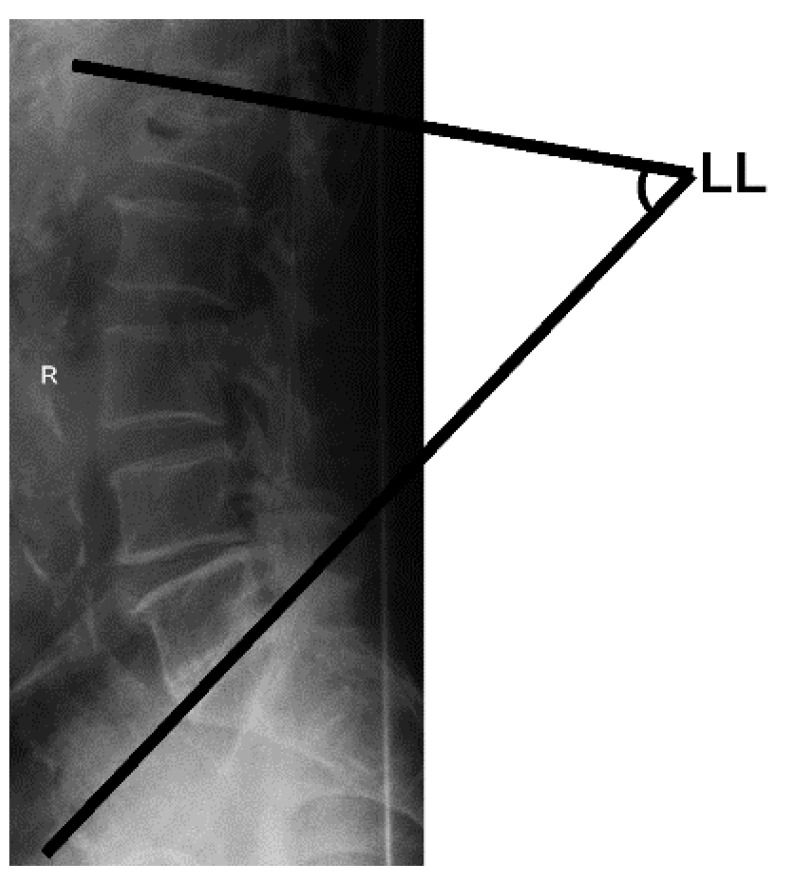
Total lumbar lordosis (LL) measurement.

To measure the lumbar lordosis angle, each participant was asked to stand with their legs and upper body in a straight line, with their eyes forward and their shoulders flexed at 90°. A lateral radiograph was taken with the cassette at a distance of 1.5 m. The angle between intersecting lines drawn from the upper endplate of the first lumbar vertebra and the upper endplate of the first sacral vertebra comprised the lumbar lordosis angle in the sagittal plane, and the calculated angle determined the severity of curvature, as shown in Figure 4 [31,32,33].

#### 2.2.4. Measurement of the Two-Dimensional Calcaneal Valgus Angle

**Figure 5:**
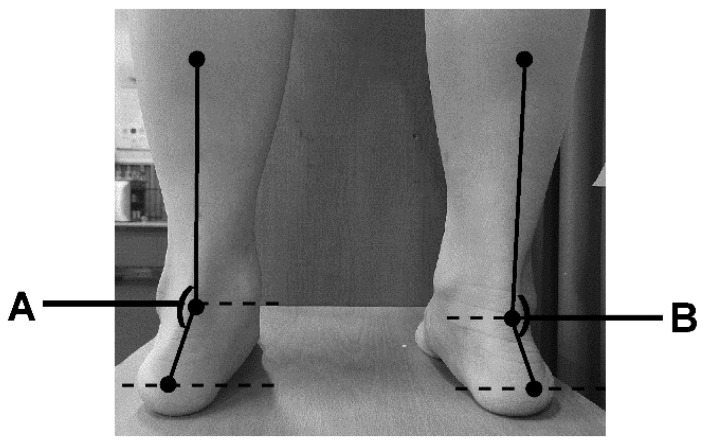
Calcaneal valgus angle measurement [A: Left calcaneal valgus angle (°), B: Right calcaneal valgus angle (°)].

To determine the calcaneal valgus angle, a plumb line test was performed, as shown in Figure 5, using the body balance index system (Exbody Inc., Seoul, Korea) [34]. The angle was measured at the intersection between the plumb line and the calcaneal tuberosity in the coronal plane.

#### 2.2.5. The Schroth Rehabilitation Exercise Program

The 12-week SRE program consisted of a warm-up exercise, the main exercise, and a cool-down period, as described in Table 2 [28].

The stages comprised the following:

- Recognition of stable breathing and normal joint range of motion (weeks 0–2)
- This stage developed symmetric movement by focusing on muscles associated with scoliosis. The patient performed stretching exercises for the full range of motion of all joints and muscles.
- Normalization of sagittal alignment [3-D Schroth application (weeks 3–8)]
- This stage corrected the hyperlordotic or hyperkyphotic state by normalizing sagittal alignment using the SRE program.
- Maintenance stage (weeks 9–12)

The SRE program was conducted by an exercise therapist with more than 5 years of clinical experience within the rehabilitation clinic (five times/week, 12 weeks), focusing on 3D self-correction and normalization of daily life through postural correction exercises.

#### 2.2.6. Data Processing

Data were analyzed using SPSS PC for Windows (version 15.0) software, and the measured values for each item are shown using descriptive statistics (mean ± standard error). Participants were classified into three groups according to the Cobb’s angle to identify whether the exercise program was effective, and analysis of covariance (ANCOVA) was applied by setting prior examination scores as covariates. The statistical processing sequence calculated the mean and standard deviation for the variables related to scoliosis. After classifying the participants into the three groups according to the severity of their scoliosis, a dependent T-test was performed before and after the exercise to verify the main effect of exercise. In addition, to examine whether the effect of exercise differed according to the degree of scoliosis, participants were classified into three groups according to the degree of scoliosis, and the normality (Shapiro–Wilk test) of each variable and the equality of variance (Levene’s test) were assessed; the pretest score of each variable was set as a covariate, and ANCOVA was performed. Statistical significance (α) was set at 0.05.

## 3. Results

### 3.1. Cobb’s Angle

Table 3 and Table 4 show the Cobb’s angle results and changes before and after SRE in each group. The model of covariate analysis for the Cobb’s angle was appropriate (F = 126.38, *p* < 0.001, η_p_^2^ = 0.870), and could explain approximately 87% of the Cobb’s angle (R^2^ = 0.864). There was a significant difference (F = 71.21, *p* < 0.001, η_p_^2^ = 0.56) in the pretest score set as a covariate, but there were no significant differences between groups (F = 0.40, *p* = 0.672). The effect size was also large with a pretest score of 0.560, while the difference between the groups was very small at 0.01. Therefore, considering the Cobb’s angle value before SRE treatment, the effect of SRE on Cobb’s angle was found to be the same in all groups.

### 3.2. Scoliometer Readings

Table 5 and Table 6 show the scoliometer results and changes before and after SRE in each group. The model of covariate analysis for the scoliometer reading was appropriate (F = 45.22, *p* < 0.001, η_p_^2 =^ 0.71), and could explain approximately 70% of the Cobb’s angle (R^2^ = 0.692). There was a significant difference (F = 72.42, *p* < 0.001, η_p_^2^ = 0.56) in the pretest score set as a covariate, but there was no difference between groups (F = 1.31, *p* = 0.279). The effect size was also large, with a pretest score of 0.56, while the difference between the groups was very small, with a difference of 0.04. Therefore, considering the scoliometer reading values before SRE treatment, the effect of SRE on the scoliometer reading was found to be the same in all groups.

### 3.3. Lumbar Lordosis Assessment

Table 7 and Table 8 show the lumbar lordosis results and changes before and after SRE in each group. The model of covariate analysis for lumbar lordosis was appropriate (F = 38.31, *p* < 0.001, η_p_^2^ = 0.67), and the model could explain approximately 67% of lumbar lordosis (R^2^ = 0.655). There was a significant difference (F = 111.06, *p* < 0.001, η_p_^2^ = 0.66) in the pretest score set as a covariate, but there was no difference between groups (F = 0.87, *p* = 0.426). The effect size was also large, with a pretest score of 0.66. The between-group difference was 0.03, which was very small. Therefore, considering the lumbar lordosis value before SRE treatment, the effect of SRE on lumbar lordosis was found to be the same in all groups.

### 3.4. Changes in the Left Calcaneal Valgus Angle

Table 9 and Table 10 show the left calcaneal valgus angle findings before and after SRE in each group. The model of covariate analysis for the left calcaneal valgus angle was appropriate (F = 10.872, *p* < 0.001, η_p_^2^ = 0.368), and could explain approximately 87% of the left calcaneal valgus angle (R^2^ = 0.334). There was a significant difference (F = 31.444, *p* < 0.001, η_p_^2^ = 0.360) in the pretest score set as a covariate, but there was no difference between groups (F = 1.145, *p* = 0.326). The effect size was also large, with a pretest score of 0.360, while the difference between the groups was very small at 0.039. Therefore, considering the left calcaneal valgus angle value before SRE treatment, the effect of SRE on the left calcaneal valgus angle was found to be the same in all groups.

### 3.5. Change in the Right Calcaneal Valgus Angle

Table 11 and Table 12 show the right calcaneal valgus angle results and changes before and after SRE in each group. The model of covariate analysis for the right calcaneal valgus angle was appropriate (F = 45.22, *p* < 0.001, η_p_^2^ = 0.71), and could explain approximately 33% of the right calcaneal valgus angle (R^2^ = 0.334). There was a significant difference (F = 9.231, *p* < 0.001, η_p_^2^ = 0.331) in the pretest score set as a covariate, but there was no difference between groups (F = 1.666, *p* = 0.198). The effect size was also large, with a pretest score of 0.300, while the difference between the groups was very small at 0.056. Therefore, the effect of SRE on the right calcaneal valgus angle was found to be the same in all groups.

## 4. Discussion

This study evaluated the factors related to the closed kinetic chain to investigate the positive effects of exercise in patients with adolescent idiopathic scoliosis. These factors included the Cobb’s angle, which is a key indicator of scoliosis after SRE intervention, and scoliometer readings, lumbar lordosis, and calcaneal valgus angle, which are related to body stability. The Cobb’s angle is classified according to the degree of scoliosis: curves <25° are considered mild scoliosis, values between 25° and 45° are moderate, and curves ≥45° are severe [35]. Particularly, a spine curvature of >30° at the end of the growth phase significantly increases the risk of complications in adulthood, including pain, chest and shoulder girdle deformities, reduced quality of life (QOL), various physical disabilities, and major respiratory problems [35,36]. Previous studies have reported that SRE had a significant effect on the treatment of scoliosis [37]. As a result of applying short-term rehabilitation composed of SRE for 7 days to 34 patients with adolescent idiopathic scoliosis with an average Cobb’s angle of 28.7 degrees, the angle of trunk rotation significantly decreased from 11.5 to 8.4 degrees, and the thoracic curves significantly decreased from 8.9 degrees to 6.5 degrees [28]. In this study, there were significant changes in the Cobb’s angle, which is the most important indicator of scoliosis diagnosis, in all three groups compared to that before the SRE intervention. In particular, the most significant change was in the moderate scoliosis or higher group with a Cobb’s angle between 30° and 39°. Additionally, the scoliometer readings and lumbar lordosis, which are indirect indicators of the Cobb’s angle, significantly improved after the 12-week SRE program.

SRE is proposed as a special program used to treat idiopathic scoliosis in young adolescents [17]. Fusco et al. [37] reported that a meta-analysis of SRE showed significant improvement in back muscle strength and respiratory function in patients with scoliosis, as well as slowed curve progression and a reduced Cobb’s angle. Moreover, in addition to improving various elements of scoliosis, function, and QOL, Schreiber et al. [12] reported that SRE is very useful for adolescents with a curve between 10° and 45°, and is very effective for psychological stability as it greatly increases self-esteem. The variables ultimately evaluated in this study were to confirm the possibility of restoring correct posture; the change of Cobb’s angle through SRE was assessed through scoliometer readings and lumbar lordosis, and posture was confirmed by the calcaneal valgus angle.

Rib humps and posterior thoracic deformities were partially reduced with a rotational breathing treatment. As the mechanisms involved in lumbar and thoracic movement on lateral bending differ, this difference may be a significant indicator when analyzing changes in the severity of scoliosis. Additionally, Schreiber et al. [38] reported that posture balance and signs and symptoms of scoliosis were improved after 6 months of SRE in adolescent patients with idiopathic scoliosis of 10° to 45°, regardless of orthosis usage. The patients’ conditions improved despite the Cobb’s angle not improving beyond the generally accepted threshold of 5°. In our study, positive changes in scoliometer readings, lumbar lordosis, and calcaneal valgus angle were observed in all groups after SRE regardless of the severity of scoliosis. Therefore SRE led to a basic improvement of the Cobb’s angle, improvement of normal postural alignment, static/dynamic postural control, and correction of spinal stability. Our findings align with the ultimate goal of SRE reported by Schreiber et al. [12], which is to improve QOL through sensory movement, posture recovery, and breathing exercises.

In this study, Schroth 3-D motion was shown to delay an additional increase in curvature and to increase the structural stability of the body, as reported in a previous study [39]. Meanwhile, a significant correlation was reported between idiopathic scoliosis (with a Cobb’s angle 15–60°) and the tibio-calcaneal angle in a previous study on the correlation between scoliosis and physical alignment in 70 adolescents [40]. Postural asymmetry is also associated with a risk of progression to idiopathic scoliosis. The curvature of scoliosis can generally be viewed as a difference in the left and right anatomical calcaneal valgus angle and leg length [41], which is consistent with the difference in the calcaneus valgus angle found in participants in the SRE program in this study. Rehabilitative exercise has been considered to have a positive effect on body balance recovery by reducing the deviation in spinal curvature angles and in lower extremity malalignment. Generally, patients with scoliosis have significantly less balancing ability when standing than those without scoliosis [42]. In terms of the pathogenesis of scoliosis, the spine is flexed to one side as the 3-D deformation of the spine occurs due to an imbalance in the foot, such as when the body load is generally concentrated on one foot. Therefore, it has been reported that a change in the angle of the foot that then supports the weight of both feet is a factor that can affect the prognosis of the disease. [43]. Balance is the ability to maintain a center of gravity on the base of support in a given environment and is an essential factor for spinal movement and stability [44]. Szulc et al. [45] reported that in patients with only right-sided thoracic scoliosis, more weight was loaded on their right side than on their left. The evaluation of the foot’s calcaneal angle is a method for assessing postural stability and is a validated tool to measure the structure of the foot based on static equilibrium [46]. The normal range of the calcaneal angle is 2°–8° of calcaneal varus/inversion [47]. However, Gauchard et al. [48] concluded that the weight load in patients with scoliosis could vary according to the level, extent, and type of curvature. Unlike previous studies, this study showed significant changes in the calcaneal valgus angle after exercise using a two-dimensional analysis system. In this study, after applying the same program to all groups, the calcaneal valgus angle returned to normal in all three groups.

Balance control is achieved through complex exercise control with inertial response and nervous system processing, and a change in sensory information immediately affects balance control [49]. In one study, 0.5% postural body sway was observed in individuals without scoliosis, as opposed to 14.5% in patients with scoliosis [50]. In a 4-month study involving trunk rotation exercise in 12 adolescent males and females with idiopathic scoliosis, Mooney et al. [51] reported that asymmetric muscle strength completely recovered, whereas balance and Cobb’s angles significantly worsened. An exercise program should be individualized according to the type and extent of scoliotic curvature. Neumann reported that thoracic and lumbar axis rotating mechanisms differ from a kinematics perspective [34]. Tension around the lumbar vertebrae transmits structural instability to the upper thoracic spine, and this abnormal spinal tension generates cross-stress effects around the thoracic spine, leading to secondary rotational displacement scoliosis. However, thoracic torsion generally occurs in the opposite direction to generate cross balance with lumbar torsion [52], which supports the premise that the type and magnitude of scoliosis curvature require different exercise programs [53]. Although SRE is based on the five principles of axis elongation, reverse bending, reverse rotation, facilitation, and stabilization, the characteristics and merits of each exercise differ. In this study, the SRE program did not apply traditional traction therapy, as SRE routines consolidate the breathing method and address inactive muscles, and a 3-D exercise for spinal deformity and scoliosis was applied using a rib as a lever. The program in this study also applied aerobic capacity improvement through a fixed bicycle exercise, lumbopelvic stabilization through a muscle cylinder exercise, and stabilization of each segment of the spine through isometric exercise. Lastly, SRE had a positive effect on vertebral rotation and scoliosis curvature and may have improved the asymmetric thoracic-lumbar rotation angle and vertebral deviation due to simultaneous reverse bending and rotation during exercise.

Passive treatment such as the use of a scoliosis brace has limitations in achieving high muscle strength improvement and normal balance [54]. Thus, in adolescent patients with idiopathic scoliosis, active rehabilitation exercises such as SRE can improve scoliometer readings, lumbar lordosis, and the calcaneal valgus angle, thereby enhancing spinal muscle function and balance ability. Furthermore, it is thought that positive effects can be obtained in daily life and sports activities. One limitation of this study was that we could not accurately measure the change in mobility due to rib asymmetry and scoliosis, and failed to evaluate the difference in the ventilation capacity of the lungs due to reduced mobility. In addition, to verify the effect more clearly, the SRE group was compared with the control group treated only with conservative treatment. However, an SRE group including various variables that can evaluate the angle of trunk rotation, Cobb’s angle, Risser sign, the angle of spine rotation on X-ray, and QOL is required. Future studies need to identify these physiological factors and consider the variables evaluated in this study.

## 5. Conclusions

SRE led to definite positive changes in the Cobb’s angle, scoliometer readings, lumbar lordosis, and the calcaneal valgus angle, regardless of the severity of scoliosis. SRE is a scoliosis-specific modality used to treat idiopathic scoliosis in young adolescents. SRE should be recommended along with conservative treatments, such as wearing aids, for the treatment of idiopathic scoliosis in adolescent patients.

## Figures and Tables

**Table 1:** Participants’ physical characteristics.

Group	Age (Year)	Height (cm)	Weight (kg)	Body Mass Index (kg/m^2^)
G1	16.15 ± 0.52	164.75 ± 2.43	54.40 ± 2.85	19.76 ± 0.50
G2	15.75 ± 0.55	164.35 ± 1.52	50.85 ± 1.89	18.72 ± 0.46
G3	14.65 ± 0.50	159.80 ± 1.12	46.60 ± 1.36	18.25 ± 0.51

G1, mild idiopathic scoliosis group with a Cobb’s angle of 10–19° (*n* = 20); G2, moderate idiopathic scoliosis group with a Cobb’s angle of 20–29° (*n* = 20); G3, severe idiopathic scoliosis group with a Cobb’s angle of ≥30° (*n* = 20).

**Table 2:** Rehabilitation exercise program for patients with scoliosis.

	Item	Program Periods (Week)					
		0–2	3–4	5–6	7–8	9–10	11–12
	Warm-up ^1^	10 min	10 min	10 min	10 min	10 min	10 min
Schroth	Cycling ^2^	10 min	15 min	20 min	20 min	25 min	25 min
	Pelvic overcorrection ^3^	15 min	20 min	25 min	-	-	-
	Rotational angular breathing ^4^	10 min	10 min	10 min	10 min	10 min	10 min
	3-D postural correction exercise ^5^	15 min	15 min	15 min	15 min	15 min	15 min
	Muscle cylinder exercise ^6^	10 min	10 min	10 min	10 min	10 min	10 min
	Isometric stabilization exercises ^7^	-	-	-	15 min	20 min	25 min
	Cool-down ^1^	10 min	10 min	10 min	10 min	10 min	10 min
	Catwalk ^8^	5 min	5 min	5 min	5 min	5 min	5 min

^1^ Stretching exercise in range without hyperflexion or pain; ^2^ Target heart rate of HRmax at 70% and at a knee joint angle of ≤ 20°; ^3^ Make proper pelvic positional corrections; ^4^ Move spine and ribs into best possible posture; ^5^ Shoulder retraction with simultaneous alignment of the sagittal profile; ^6^ Shift of the shoulder girdle against the pelvic girdle on the wall-bar; ^7^ Diagonal pulling of the hip in coordination with the concave side; ^8^ Asymmetric exercise to improve postural correction and posture during activities of daily life.

**Table 3:** Cobb’s angle readings in each group.

Group	Before	After	Δdiff.	t-Value	Degrees of Freedom
G1	15.50 ± 2.78	9.90 ± 3.61	−5.60 ± 2.42 ***	10.371	19
G2	24.60 ± 3.25	18.60 ± 5.04	−6.00 ± 4.48 ***	5.984	19
G3	39.35 ± 7.57	30.40 ± 7.32	−8.95 ± 4.11 ***	9.739	19
Total	24.68 ± 11.07	19.63 ± 10.07	−6.85 ± 4.01 ***	13.23	59

G1, Idiopathic scoliosis group with Cobb’s angle of 10–19° (*n* = 20); G2: Idiopathic scoliosis group with Cobb’s angle of 20–29° (*n* = 20); G3: Idiopathic scoliosis group with a Cobb’s angle of ≥30° (*n* = 20); Δdiff., mean difference; ***, *p* < 0.001.

**Table 4:** Analysis of covariance results for Cobb’s angle.

Item	Source	Sum of Squares	Degrees of Freedom	Mean Square	F	Significance	η_p_^2^
Cobb’s angle	Corrected Model	5213.82 ^a^	3	1737.94	126.38	<0.001	0.870
	Intercept	9.88	1	9.88	0.72	0.400	0.010
	Covariance	979.28	1	979.28	71.21	<0.001	0.560
	Group	11.00	2	5.50	0.40	0.672	0.010
	Error	770.12	56	13.75			
	Total	29,112.00	60				
	Corrected Total	5983.93	59				

^a.^ R^2^ = 0.871 (adjusted R^2^ = 0.864).

**Table 5:** Scoliometer readings in each group.

Group	Before	After	Δdiff.	t-Value	Degrees of Freedom
G1	7.00 ± 2.10	4.70 ± 2.16	−2.30 ± 1.78 ***	5.779	19
G2	8.70 ± 2.66	6.60 ± 2.44	−2.10 ± 1.62 ***	5.801	19
G3	12.70 ± 2.76	8.70 ± 2.56	−4.00 ± 1.81 ***	9.903	19
Total	9.47 ± 3.45	6.67 ± 2.86	−2.80 ± 1.91 ***	11.34	59

G1, Idiopathic scoliosis group with a Cobb’s angle of 10–19° (*n* = 20); G2: Idiopathic scoliosis group with a Cobb’s angle of 20–29° (*n* = 20); G3: Idiopathic scoliosis group with a Cobb’s angle of ≥30° (*n* = 20); Δdiff., mean difference; ***, *p* < 0.001.

**Table 6:** Analysis of covariance results for scoliometer readings.

Item	Source	Sum of Squares	Degrees of Freedom	Mean Square	F	Significance	η_p_^2^
**Scoliometer reading**	Corrected Model	343.52 ^a^	3	114.51	45.22	<0.001	0.71
	Intercept	0.02	1	0.02	0.01	0.934	<0.001
	Covariance	183.39	1	183.39	72.42	<0.001	0.56
	Group	6.62	2	3.31	1.31	0.279	0.04
	Error	141.81	56	2.53			
	Total	3152.00	60				
	Corrected Total	485.33	59				

^a.^ R^2^ = 0.708 (Adjusted R^2^ = 0.692).

**Table 7:** Lumbar lordosis on the sagittal plane according to group.

Group	Before	After	Δdiff.	t-Value	Degrees of Freedom
G1	40.30 ± 6.47	44.85 ± 5.50	4.55 ± 4.38 ***	4.643	19
G2	39.30 ± 5.85	43.95 ± 5.20	4.65 ± 3.53 ***	5.894	19
G3	39.15 ± 7.87	42.65 ± 7.33	3.50 ± 3.94 ***	3.972	19
Total	39.58 ± 6.68	43.82 ± 6.04	4.23 ± 3.93 ***	8.330	59

G1, Idiopathic scoliosis group with a Cobb’s angle of 10–19° (*n* = 20); G2: Idiopathic scoliosis group with a Cobb’s angle of 20–29° (*n* = 20); G3: Idiopathic scoliosis group with a Cobb’s angle of ≥30° (*n* = 20); Δdiff., mean difference; ***, *p* < 0.001.

**Table 8:** Analysis of covariance results for lumbar lordosis.

Item	Source	Sum of Squares	Degrees of Freedom	Mean Square	F	Significance	η_p_^2^
Lumbar lordosis	Corrected Model	1450.35 ^a^	3	483.45	38.31	<0.001	0.67
	Intercept	360.75	1	360.75	28.59	<0.001	0.34
	Covariance	1401.42	1	1401.42	111.06	<0.001	0.66
	Group	21.90	2	10.95	0.87	0.426	0.03
	Error	706.63	56	12.62			
	Total	11,7351.00	60				
	Corrected Total	2156.98	59				

^a.^ R^2^ = 0.672 (Adjusted R^2^ = 0.655).

**Table 9:** The degree of change in the left calcaneal valgus angle in each participant group.

Group	Before	After	Δdiff.	t-Value	Degrees of Freedom
G1	9.55 ± 3.68	6.20 ± 3.49	−3.35 ± 2.88 ***	5.06	19
G2	9.50 ± 4.31	6.25 ± 3.97	−3.25 ± 2.88 ***	5.40	19
G3	10.10 ± 2.55	5.40 ± 2.80	−4.70 ± 3.57 ***	5.88	19
Total	9.72 ± 3.53	5.95 ± 3.41	−3.76 ± 3.17 ***	9.21	59

G1, Idiopathic scoliosis group with a Cobb’s angle of 10–19° (*n* = 20); G2: Idiopathic scoliosis group with a Cobb’s angle of 20–29° (*n* = 20); G3: Idiopathic scoliosis group with a Cobb’s angle of ≥30° (*n* = 20); Δdiff., mean difference; ***, *p* < 0.001.

**Table 10:** Analysis of covariance results concerning the left calcaneal valgus angle.

Item	Source	Sum of Squares	Degrees of Freedom	Mean Square	F	Significance	η_p_^2^
Left calcaneal valgus angle	Corrected Model	253.532 ^a^	3	84.511	10.872	<0.001	0.368
	Intercept	0.804	1	0.804	0.103	0.749	0.002
	Covariance	244.432	1	244.432	31.444	<0.001	0.360
	Group	17.799	2	8.900	1.145	0.326	0.039
	Error	435.318	56	7.774			
	Total	2813.000	60				
	Corrected Total	688.850	59				

^a.^ R^2^ = 0.368 (adjusted R^2^ = 0.334).

**Table 11:** The degree of change in the right calcaneal valgus angle in each participant group.

Group	Before	After	Δdiff.	t-Value	Degrees of Freedom
G1	9.50 ± 4.92	7.20 ± 3.33	−2.30 ± 3.57 **	2.88	19
G2	9.35 ± 4.93	5.50 ± 3.30	−3.85 ± 3.77 ***	4.56	19
G3	8.45 ± 3.65	6.10 ± 3.51	−2.35 ± 4.37 *	2.43	19
Total	9.10 ± 4.48	6.27 ± 3.40	−2.83 ± 3.90 ***	5.62	59

G1, Idiopathic scoliosis group with a Cobb’s angle of 10–19° (*n* = 20); G2: Idiopathic scoliosis group with a Cobb’s angle of 20–29° (*n* = 20); G3: Idiopathic scoliosis group with a Cobb’s angle of ≥30° (*n* = 20); Δdiff., mean difference; ***, *p* < 0.001; **, *p* < 0.01; *, *p* < 0.05.

**Table 12:** Analysis of covariance results concerning right calcaneal valgus angle.

Item	Source	Sum of Squares	Degrees of Freedom	Mean Square	F	Significance	η_p_^2^
Right calcaneal valgus angle	Corrected Model	225.575 ^a^	3	75.192	9.231	<0.001	0.331
	Intercept	74.636	1	74.636	9.163	0.004	0.141
	Covariance	195.842	1	195.842	24.042	<0.001	0.300
	Group	27.147	2	13.574	1.666	0.198	0.056
	Error	456.158	56	8.146			
	Total	3038.000	60				
	Corrected Total	681.733	59				

^a.^ R^2^ = 0.331 (adjusted R^2^ = 0.295).

## Data Availability

The data presented in this study are available upon request from the authors. The data are not publicly available owing to privacy and ethical restrictions.
